# A mobile app for chronic disease self-management for individuals with low health literacy: A multisite randomized controlled clinical trial

**DOI:** 10.3390/jal4020005

**Published:** 2024-04-30

**Authors:** Raymond L Ownby, Michael Simonson, Joshua Caballero, Kamila Thomas-Purcell, Rosemary Davenport, Donrie Purcell, Victoria Ayala, Juan Gonzlez, Neil Patel, Kofi Kondwani

**Affiliations:** 1 Department of Psychiatry and Behavioral Medicine, Nova Southeastern University, Fort Lauderdale FL; 2 Instructional Technology and Distance Education Program, Fischler College of Education, Nova Southeastern University, Fort Lauderdale FL; 3 University of Georgia, Athens GA; 4 College of Health Sciences, Nova Southeastern University, Fort Lauderdale FL; 5 Department of Psychiatry and Behavioral Medicine, Nova Southeastern University, Fort Lauderdale FL; now at Satcher Health Leadership Institute, Morehouse School of Medicine, Atlanta GA; 6 Department of Community Health & Preventive Medicine, Morehouse School of Medicine, Atlanta GA FL

**Keywords:** Health literacy, chronic disease self-management, patient activation, quality of life, medication adherence

## Abstract

The purpose of this study was to evaluate the effects of a mobile app designed to improve chronic disease self-management in patients 40 years and older with low health literacy and who had at least one chronic health condition, and to assess the impact of delivering information at different levels of reading difficulty. A randomized controlled trial was completed at two sites. Individuals aged 40 years and older screened for low health literacy who had at least one chronic health condition were randomly assigned to a tailored information multimedia app with text at one of three grade levels. Four primary outcomes were assessed: patient activation, chronic disease self-efficacy, health-related quality of life, and medication adherence. All groups showed overall increases in activation, self-efficacy, and health-related quality of life, but no change in medication adherence. No between-group differences were observed. The mobile app may have been effective in increasing participants’ levels of several psychosocial variables, but this interpretation can only be advanced tentatively in light of lack of control-experimental group differences. Reading difficulty level was not significantly related to outcomes.

## 1. Introduction

Health literacy is the degree to which individuals have the ability to find, understand, and use information and services to inform health-related decisions and actions for themselves and others [1]. It is related to health status and health outcomes across a wide range of contexts and health conditions. The 2003 United States (US) National Assessment of Adult Literacy showed that more than 75 million Americans had only basic health literacy skills, indicating that 1 in 4 Americans have problems understanding information about healthcare [2]. More recent studies of literacy and numeracy skills in the general population suggest that the situation has not changed [3,4]. Research also shows that the problem is not limited to the US, with similar findings in Canada, Europe and Africa [5-7].

Further, health literacy is lower in persons from racial and ethnic minoritized groups as well as persons 40 years of age and older [2,8] and may be an important factor in health disparities [9,10]. In the US, 24% of Black persons (9.5 million) and 41% of Hispanic persons (21 million) have below basic levels of health literacy [2]. Members of minoritized groups have lower levels of health literacy and compelling evidence links race and ethnicity to disparities in health via health literacy [11-18]. Members of racially minoritized groups and persons in middle age or older are also more frequently affected by chronic diseases such as cancer, high blood pressure, heart attack, stroke, diabetes, asthma, hepatitis, HIV infection, mental health disorders and many others. The twin burdens of chronic disease and low levels of health literacy thus fall disproportionately on those most in need – members of minorities and persons in middle age or older, all of whom may experience one or more chronic conditions while not having the health literacy skills they need to cope [9,19]. Interventions to improve health literacy are thus clearly needed.

Providing information for patients in clinical settings on self-management of their health conditions may be a useful strategy in addressing low health literacy. In the traditional methods that use pamphlets or handouts supported by conversation in a brief clinical encounter, however, information is often not actually read or remembered [20,21], and recommendations may not be implemented among people diagnosed with chronic conditions [22,23].

One strategy to increase the impact of such information on patient behavior is tailoring. Tailoring information, defined as using various methods to create individualized communications for patients [24] aims to reduce the burden of self-management on health consumers by giving them useful information that is relevant to their needs or concerns and that they can understand and use. Various information interventions have been developed to improve patients’ health literacy [12,25,26], including matching health education content to patient characteristics and tailoring health messaging to make it more directly relevant to patients. While these techniques have often been successful [25,27], creating individually-tailored health information is labor intensive and thus may not be widely available.

A possible solution to the problem of giving patients the information they want and need in a form they can use has been the development of computer-based interventions to automate message tailoring [28,29]. Computer-based tailoring creates the possibility that high-quality individualized health information can be made available to those who need it. Information can be delivered to patients when they want it and it can target content they are interested in, a process of providing precision health information [30]. Analogous to the processes underlying the precision medicine approach to the somatic treatment of diseases [31], computer-based tailoring can take a patient’s personal characteristics, including their expressed concerns or problems, and provide detailed information to help them understand their health conditions and develop self-management skills.

A potentially critical variable in the tailoring process is ensuring that content is appropriate to the patient’s level of health literacy. If information is not understood, even if tailored, it may not impact patients’ behavior. Studies show that while experts recommend that materials for patients be written at a 4th or 6th grade level and match patients’ level of health literacy [32-34], multiple studies show that most patient education materials are at much more difficult levels [35-39].

Similarly, while interventions have been developed to improve health literacy [40,41] they are difficult to scale to levels needed to meet the challenge of low health literacy (millions of persons worldwide) due to their cost [29,41,42]. Effective interventions with the potential for wider dissemination at reasonable costs are urgently needed. For many of the problems that are the focus of CDSM, well-defined behavioral strategies exist for their management (e.g., cognitive behavioral therapy for sleep [43] or mood problems [44]) holding the possibility that effective tailoring might help patients develop relevant knowledge and behavioral skills.

Assessments of CDSM programs have focused on several outcomes, and in this project, we focused on four that we judged were most relevant to the effects of a CDSM-targeting intervention and that would allow us to compare our results with those of other researchers. *Patient activation* [45] is defined as the extent to which they are actively involved in their healthcare, and has been related to a number of important health status variables, including emergency department visits, receiving breast cancer screening as well laboratory measures such as hemoglobin A1C (related to diabetes) and HDL (high density lipoprotein related to cardiovascular disease risk) [46]. Activation has also been associated with self-management behaviors [47] as well as quality of life [48], and studies show that interventions that improve patient skills can increase activation [49]. Finally, activation is related to self-management behaviors [50] and changes in activation are related to changes in health outcomes [51] and healthcare costs [52].

*Self-efficacy*, or a person’s belief in his or her capacity to reach specific goals [53], is a key concept in understanding health behavior [54], especially in relation to patients’ health behavior [54] and in CDSM [55]. In-person and internet-delivered CDSM programs have a positive impact on self-efficacy [56,57] which in turn have been related to self-management [58-60]. Another highly relevant potential outcome of improved CDSM skills is improved *health-related quality of life* (HRQOL). Self-reported health status has often been studied as an outcome in CDSM studies [57,61,62], and is clearly an important aspect of improved self-management skills. Finally, *medication adherence* is an essential aspect of self-management behavior. Poor medication adherence is common in patient populations with estimates of adherence ranging from 55 [63] to 75%[64]. Low levels of adherence have been linked to numerous adverse health outcomes [65], increased healthcare costs [66], and studied as an outcome in other studies of CDSM [62] [63]

The objective of this study was to assess whether a mobile app for CDSM providing individually-tailored health information would have a positive impact on participants’ activation, self-efficacy, quality of life and medication adherence. We chose CDSM because we believed it was a logical target for a health literacy intervention. In an approach that cuts across specific diseases, CDSM targets problems and skills needed to cope with issues such as fatigue, pain, stress, depression, sleep disturbance and treatment adherence. Studies show that in-person CDSM classes improve patients’ functioning and reduce healthcare utilization [55-57,61,62,68,69] but their availability is limited due to the lack of qualified personnel, cost, and accessibility. It was also hypothesized that information presented at reading levels consistent with expert recommendations (3rd to 6th grade levels) would have a greater impact on these variables than information presented at an 8th grade level.

## 2. Materials and Methods

### 2.1 Development

In this project, we worked to address issues of providing health information to individuals with low levels of health literacy by developing a tailored information app focused on chronic disease self-management (CDSM) [61] with three versions: one with text at 8th grade level (a control condition), a second at 6th grade (an experimental group), and a third at 3rd grade level supported by audio narration (a second experimental group) [67].

Initial guidance on app content was drawn from a review of existing sources on CDSM supplemented by a qualitative study that explored patients with chronic health conditions needs for information about managing their conditions [70]. A multidisciplinary team comprising representatives from medicine, nursing, psychology, pharmacy, public health, and education was assembled to develop content. Team members with special expertise in developing culturally and ethnically appropriate education materials were included as well (KTP, KK).

App content was developed by individual team members and reviewed by the team for appropriateness of content and format as well as for the usefulness and relevance of graphic content. Once drafts of the modules were available, they were subjected to usability testing (including asking for comments on the module content as well as the format) with groups of potential users with low levels of health literacy, asking them not only for feedback on the interface and ease of use of the app but also on app content. Modules were revised and retested as needed. The app was conceived as a series of topical modules that would consist of a series of screens within the app. Each module included screens conveying an orientation to its purpose, assessment of the participants’ current status by way of questions, general health information on each topic, individually-tailored content, and a summary. Self-test questions were included to help participants understand how well they learned the module’s contents.

Information was presented as text on a series of screens, supplemented by pictures, graphics, and narrated animations consistent with the principles of multimedia learning [71]. An outline of each module’s contents is provided in Supplementary Table 1, and example screenshots pages that included suggestions about self-care through improving diet is presented and suggestions about strategies for working with the participant’s doctor are included in Supplementary Figures 1 and 2. Additional information on the development of the modules and user experience in working with them has been reported in [72]. Modules with the same content were created at three levels of reading difficulty based on the Fry [73] and Flesch Reading Ease [74,75] scores of the text they contained (3rd grade, with text narrated; 6th and 8th) using Health Literacy Advisor^®^ (Bethesda MD: Health Literacy Innovations LLC), a software plugin working with Microsoft Word^®^.

### 2.2 Participants.

Participants were recruited from participants in previous studies, from local health clinics and medical practices, and by word of mouth. At the Atlanta site, a paid recruiter visited local churches where she could screen potential participants as well as give them information about participation. Information and race and ethnicity was collected as required by the U.S. National Institutes of Health for grant recipients [76]. Race and ethnicity were self-reported by participants. Gender was also self-reported, with transgender participants considered as the gender of their chosen identity.

### 2.2 Screening.

Participants were initially screened to determine their potential eligibility using a brief interview that elicited medical history, medication use, and education. They were administered a short form of the Rapid Estimate of Adult Literacy in Medicine (REALM) [77] using a previously validated cut-off for health literacy at or below the 8th grade level [78].

### 2.3 Inclusion and exclusion criteria.

To be eligible to participate in the study, participants were required to be 40 years of age and older, have at least one chronic health condition for which they were currently treated, have an education level less than 16 (i.e., not be a college graduate), and score below the cut-off score on the short form of the REALM. Persons 40 years of age and older were included because of the increased prevalence of chronic health conditions in persons in this age range [79,80]. The criterion of having less than a college education was derived from our findings in a previous study [30] that no participants who had successfully completed a college degree had inadequate health literacy. We did not include specific criteria for level of technological skill or diversity with respect to race or ethnicity. It should be noted that while participants were assessed to have low levels of health literacy on a standard measure, they may have had some disease-specific knowledge or skills as evidenced by the fact that they had been diagnosed with a health condition.

In this study, we chose to define chronic health conditions broadly as we wished to understand not only self-management but also to take into account the observation that the most common chronic condition is multimorbidity [81]. We thus chose to cast a wide net to include conditions with varying levels of severity and likely impact on participants’ daily lives. We included all the conditions listed in Table 1, with the additional criterion that participants’ conditions had to be treated with at least one medication prescribed (or recommended in the case of over-the-counter medications) by a healthcare provider. The list was based on the conditions included in the Functional Comorbidity Index [82] as it focused on the relation of health conditions to physical function, but was expanded to include a number of conditions requiring treatment that were commonly used in US Medicare reporting [83] and other multimorbidity indexes [84] such as hypertension and dyslipidemia. We also expanded the number of mental health conditions to include psychotic and bipolar disorders as well as included HIV/AIDS and chronic fatigue syndrome/myalgic encephalomyelitis. This left what we judged would be a useful balance between a detailed assessment of patients’ status, broad inclusion criteria to support generalizability of findings, and participant assessment burden.

### 2.4 Measures

Participants completed an extensive battery of measures as part of a baseline assessment, with self-report outcome measures administered immediately after completing the intervention and then three months later. Most self-report measures were administered by computer, with questions read aloud by the interviewing software to minimize the impact of participants’ reading skill on their ability to respond to questions. At the baseline visit, participant demographic information, level of education, and medical history were assessed in an individual interview. As noted above, the medical history interview was based on the medical conditions comprising the Functional Comorbidity Scale [82] [83] but expanded to include additional health conditions common in middle aged and older adults [83]. Self-report measures were administered via audio computer-assisted self-interview software (Bethesda MD: Questionnaire Development System) that read all questions aloud to participants to keep effect of reading ability on participant responses to a minimum.

In order to provide a standardized assessment of participants’ reading skills, trained assessors individually administered the Woodcock-Johnson Psycho-Educational Battery [85] Passage Comprehension subtest. This measure provides a grade equivalent score that helped characterize participants’ reading levels. The FLIGHT/VIDAS health literacy scale was used in this study because of its desirable psychometric characteristics [86] that include a wide range of scores; other health literacy measures often have ceiling effects that reduce the range of observed scores that make them less useful in statistical analyses [87].

### 2.5 Outcomes

The four outcomes we studied were assessed with widely-used measures of each construct. Activation was evaluated with the Patient Activation Measure [45]. We used the ten-item version based on the 13-item short form that has been shown to have good validity and reliability [88,89]. Higher scores are associated with changes in patients’ engagement in medical treatment and belief in their self-management skills [49]. Self-efficacy was evaluated with the Chronic Disease Self-Efficacy Scale, used in multiple studies of CDSM programs. It also has demonstrated reliability and validity [90,91]. Higher scores are associated with increases in patients’ perceptions of their ability to lead a regular life in the face of the challenges of a chronic health condition and are inversely correlated with health distress, illness intrusiveness, and activity limitations [91]. The Medical Outcomes Study, Short Form 36 General Health subscale (MOS SF36 [92]) is one of the most widely used measures for understanding psychosocial functioning related to health. It has well-established reliability and validity for use with persons with chronic health conditions [93,94] and is related to both the physical and emotional domains assessed by the SF36 [95]. Higher scores on the indicate higher levels of perceived health, as higher scores mean that participants have responded more positively when asked to rate their health overall. In this study it is used as a measure of HRQOL.

The Gonzalez-Lu questions were included as a measure of adherence because they have been validated against electronically-recorded medication adherence and for their simplicity [96,97]. As the four questions were highly intercorrelated, we reduced them to single score via principal axis factor analysis and used resulting factor scores as a single measure. This approach avoids the limitations of simply summing a group of items [98] and has good psychometric properties [99]. Scores on this summary measure expressed as standard (z scores) have a mean of zero, with more positive scores indicating higher levels of self-reported adherence.

### 2.6 Procedures

After initial screening, potential participants were scheduled for an in-person eligibility visit when, after obtaining verbal consent, they completed measures of health literacy, reading comprehension, a hearing and vision screening, and a medical history interview to determine their eligibility. Participants’ visual acuity was assessed with the Rosenbaum Pocket Visual Screener [100], while auditory acuity was assessed by evaluating the participant’s ability to correctly respond to open-ended sentence completions problems from the Woodcock-Johnson Psycho-Educational Battery [85] heard through the computer audio, such as “A bird swims, a fish …?”. Participants were required to respond to four items to confirm their ability to understand the audio included in the modules.

Eligible persons were then scheduled for a baseline visit during which they completed self-report measures and some individually-administered measures of academic skills. At the first intervention visit, participants were randomly assigned by research assistants to one of the three groups (3rd [intervention], 6th [intervention], or 8th [control] grade reading levels) and returned to for the intervention visits. Randomization was done by permuted blocks of 3 and 6 with a list generated by an online resource (London: Sealed Envelope, Ltd). Intervention visits occurred over two to three weeks with a maximum of two sessions per week during which the participants worked through the CDSM modules for a total of three sessions. During intervention sessions, participants worked through the modules on tablet computers (Microsoft Surface Pros^®^) as preliminary work suggested that many of them would have difficulty in interacting with the modules on smaller screens.

In the first session, participants reviewed an introductory module that explained the purpose of the information, an adherence module that emphasized not only strategies for treatment adherence but also how to work with health care professionals, and a module on stress, its effects, and management techniques. In the second session, participants reviewed modules on sleep, mood, pain, and memory. Finally, in the third session, they worked with modules on fatigue, shortness of breath, and anger.

After completing the modules, participants returned within several days for the first follow-up visit during which they again responded to self-report measures and completed an individual semi-structured interview that elicited their reactions to the modules and the extent to which they had adopted any of the recommendations they contained. Three months later, participants returned for a second follow-up visit during which they again responded to self-report measures and completed the same semi-structured interview.

### 2.7 Human subjects approval

All study procedures were completed under protocols approved by the Nova Southeastern University Institutional Review Board (2018-685-NSU) and the Emory University Institutional Review Board (MODCR001-IRB00087112). All participants provided verbal consent for screening and written informed consent for all other study procedures.

### 2.8 Statistical analyses

Planned analyses assessed the study hypotheses that persons receiving the intervention would (1) show significant increases in measures of activation, chronic disease self-efficacy, health-related quality of life and medication adherence and (2) that persons receiving the information at 6th and 3rd grade levels (experimental conditions) would show greater change than those receiving the material at the 8th grade level (control condition).

Preliminary review of data and descriptive measures were obtained using SPSS version 28 (Armonk NY: IBM). Mixed effects random intercept models were evaluated with the statistical program R, version 4.2.1 [101] using the *lme4* package [102]. All models included participant age, gender, race, and site of data collection as well as time and treatment group and their interaction. Tests of study hypotheses were completed after maximum likelihood estimation using Satterthwaite approximations of *p* values [103]. Tests of the statistical significance of within- and between-group differences were obtained using the *emmeans* package with Tukey corrections for multiple comparisons [104].

Based on evaluation of between-group differences at the two sites as well as theoretical considerations, model covariates were chosen to control for likely confounders as well as observed between-site differences. They were chosen based on considerations of likely confounding impact on outcome measures (age, gender, race, and education) and observed between-site differences in level of health literacy and level of multimorbidity (FLIGHT/VIDAS health literacy scale and number of health conditions). In addition, because of observed differences in participant characteristics at the two sites, site itself was included as a covariate.

#### 2.8.1 Sample size

Target sample size was determined during the planning phase of the project using the mixed effects models simulation routine in PASS 16[105] which showed that a minimum sample size of 30 per group would provide a power greater than 0.90 to evaluate study hypotheses as the interaction of treatment group with time. Effect sizes for the analysis were based on previous observation of the effects of a similar intervention [106].

#### 2.8.2 Evaluation of minimal clinically important difference (MCID)

Although in the original protocol of the study we only planned to evaluate between and within group treatment effects based on their statistical significance alone, as post-hoc assessments of treatment effects we calculated effect sizes as Cohen’s *d* statistic [107]. We therefore provide the *d* statistic based on the chi-square-based likelihood ratio test evaluating models of increasing complexity by adding treatment effects and testing the differences between models. The resulting chi-square value was then converted to the more familiar *d* statistic using the *esc* package in R [108] facilitating comparisons with other studies evaluating chronic disease self-management interventions using this effect size measure. We also provide an interpretation of the obtained effect sizes in the context of the minimal clinically important difference (MCID) to facilitate interpretation of study results. Although direct estimates of the MCID for the outcomes were not available for all instruments, we followed the practice of others in viewing effect sizes of 0.20 to 0.50 (small to medium effects [109]) as clinically meaningful. In this report, effect sizes greater than 0.20 are interpreted as potentially clinically meaningful, while those greater than or equal to 0.50 as meeting a more rigorous criterion and being more likely to represent an MCID [109,110].

## 3. Results

**Figure 1.**
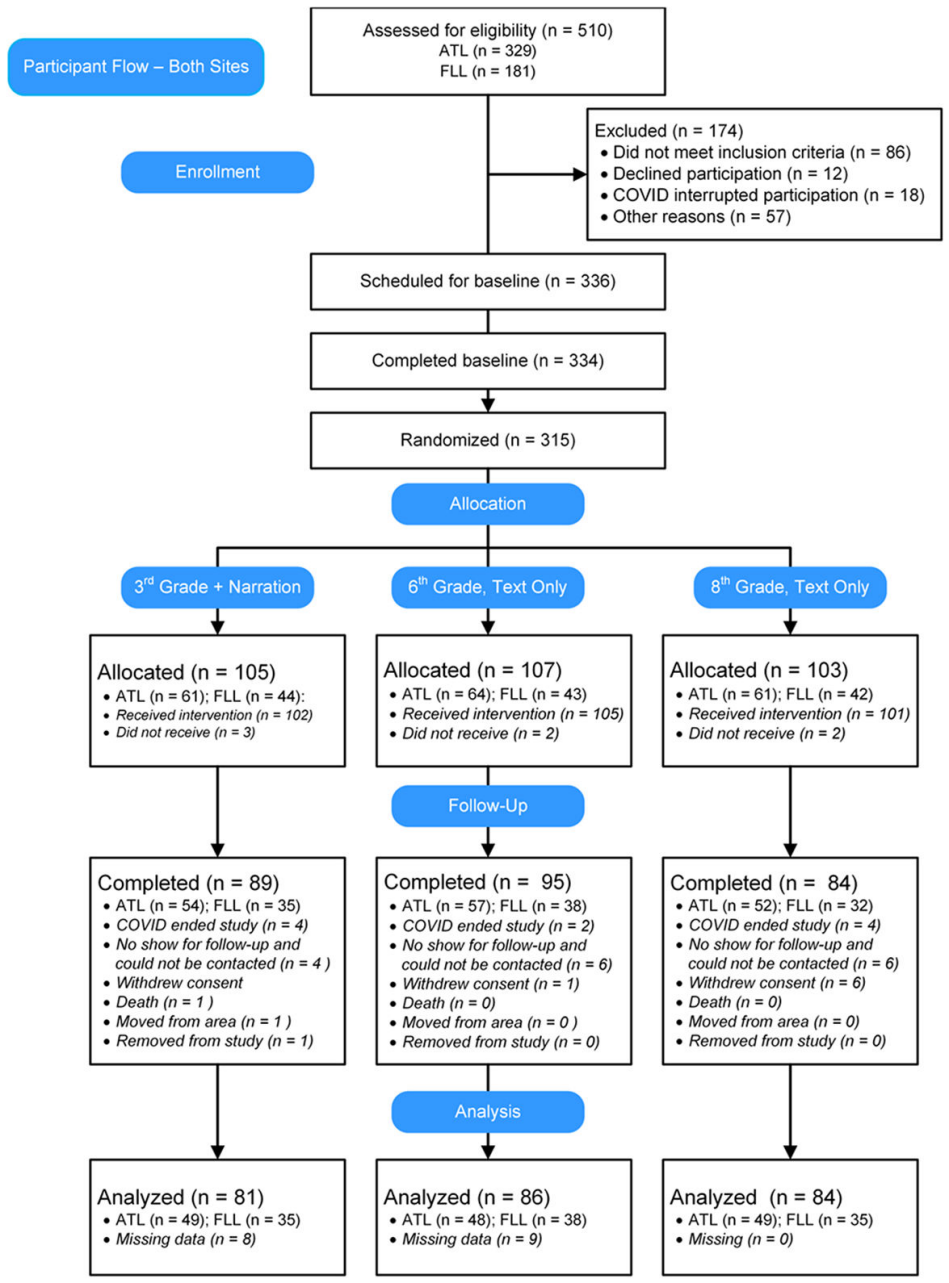
CONSORT diagram

Figure 1 presents the CONSORT diagram for participant flow during the trial for both sites combined, and Table 1 presents descriptive data for participants who completed at least one intervention session at each site and for both groups overall. Study activities began on September 20, 2018 at the Fort Lauderdale site and on October 20, 2018 in Atlanta. All study activities concluded in November, 2020. The study concluded at the end of the period of financial support provided by a government grant. Both participant gender and race were differently distributed at the two sites, with relatively more male and white participants at the Fort Lauderdale site. The two groups of participants did not vary on three of the outcome measures, although Fort Lauderdale participants reported slightly greater medication adherence although the comparison was not significant.

While 334 participants completed the baseline visit, only 315 actually completed at least the first intervention visit. Reasons for their loss included that they did not appear for the first scheduled visit and could not be contacted (n = 9), they did not comply with study procedures (n = 4), they were caregivers who could not be away from the person for whom they cared (n = 3), and their own illness (n = 3).

**Figure 2.**
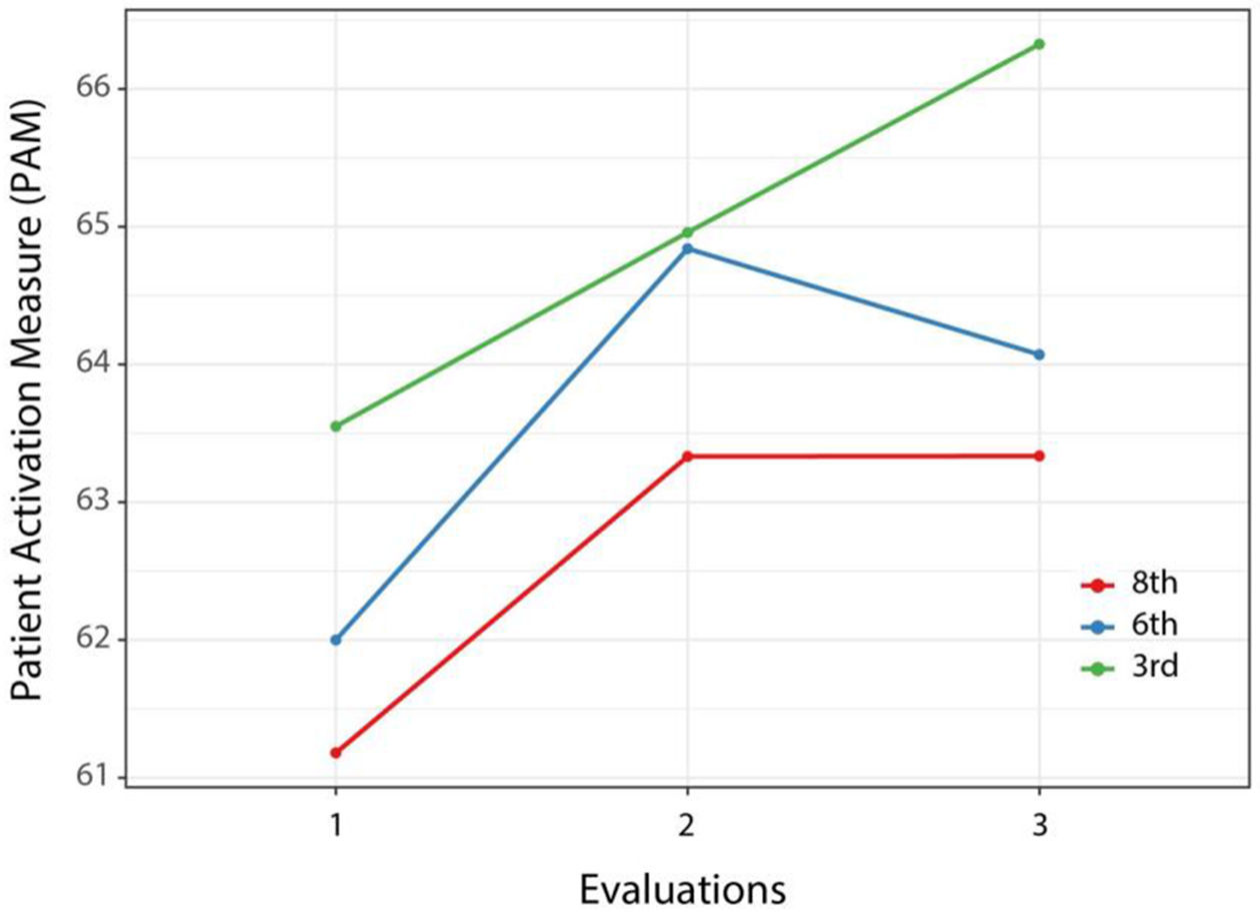
Patient Activation Measure means by group at each evaluation

The random intercept model for the Patient Activation Measure is presented in Table 2, with model-based means for each group at each time displayed in Figure 2. While the interaction of treatment group with time was not statistically significant, there was a significant effect for time, with all groups showing increases in activation after the intervention. Although level of activation appears to continue to increase between the first and second follow-up visits for the 3rd grade group, the difference between this group’s activation and the other groups was not significant (all *p*s > 0.50) and the within-subjects difference also was not significant (*p* > 0.50). Calculation of treatment effect size through the likelihood ratio test of models with and without the effect of time resulted in significant chi square (χ2 [2] = 10.27, *p* = 0.006) for the effect of time, associated with a *d* value of 0.37 (95th CI 0.14-0.60), an intermediate effect size greater than a small effect of 0.20 but not reaching the more rigorous criterion of 0.50.

**Figure 3.**
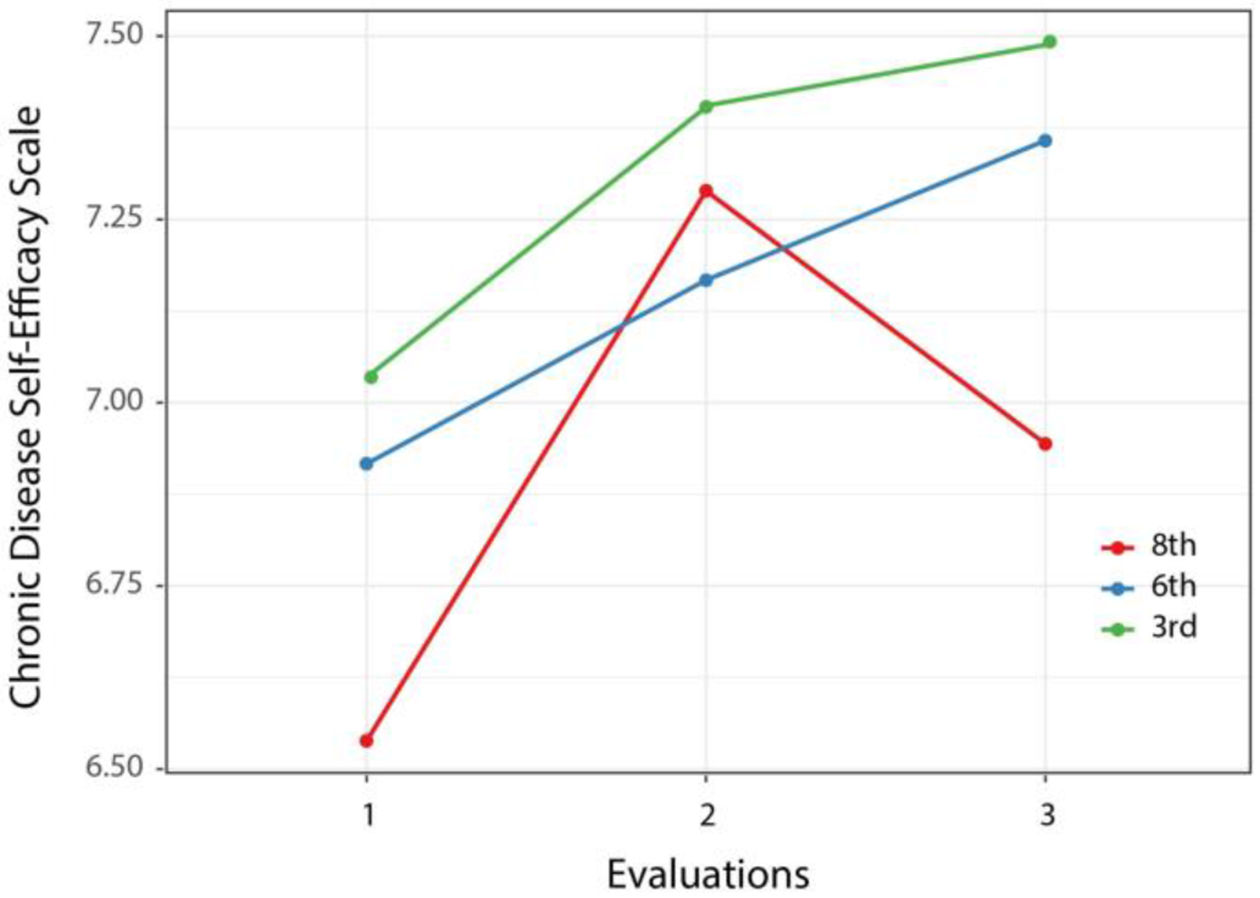
Chronic Disease Self Efficacy means by group at each evaluation

The model for the Chronic Disease Self-Efficacy Scale is presented in Table 3. Education, health literacy, and total number of health conditions as well as time were related to this outcome. Model-derived means are plotted in Figure 3. Although the level of self-efficacy appears to decline for the 8th grade group at second follow-up the difference between the 8th and 3rd grade groups (which appears to increase) was not significant (*p* = 0.14). Calculation of treatment effect size through the likelihood ratio test resulted in significant chi square (χ2 [2] = 23.64, *p* < 0.001) for the effect of time, associated with a *d* value of 0.58 (95th CI 0.34-0.81), meeting the more rigorous criterion of 0.50 for an MCID [113].

**Figure 4.**
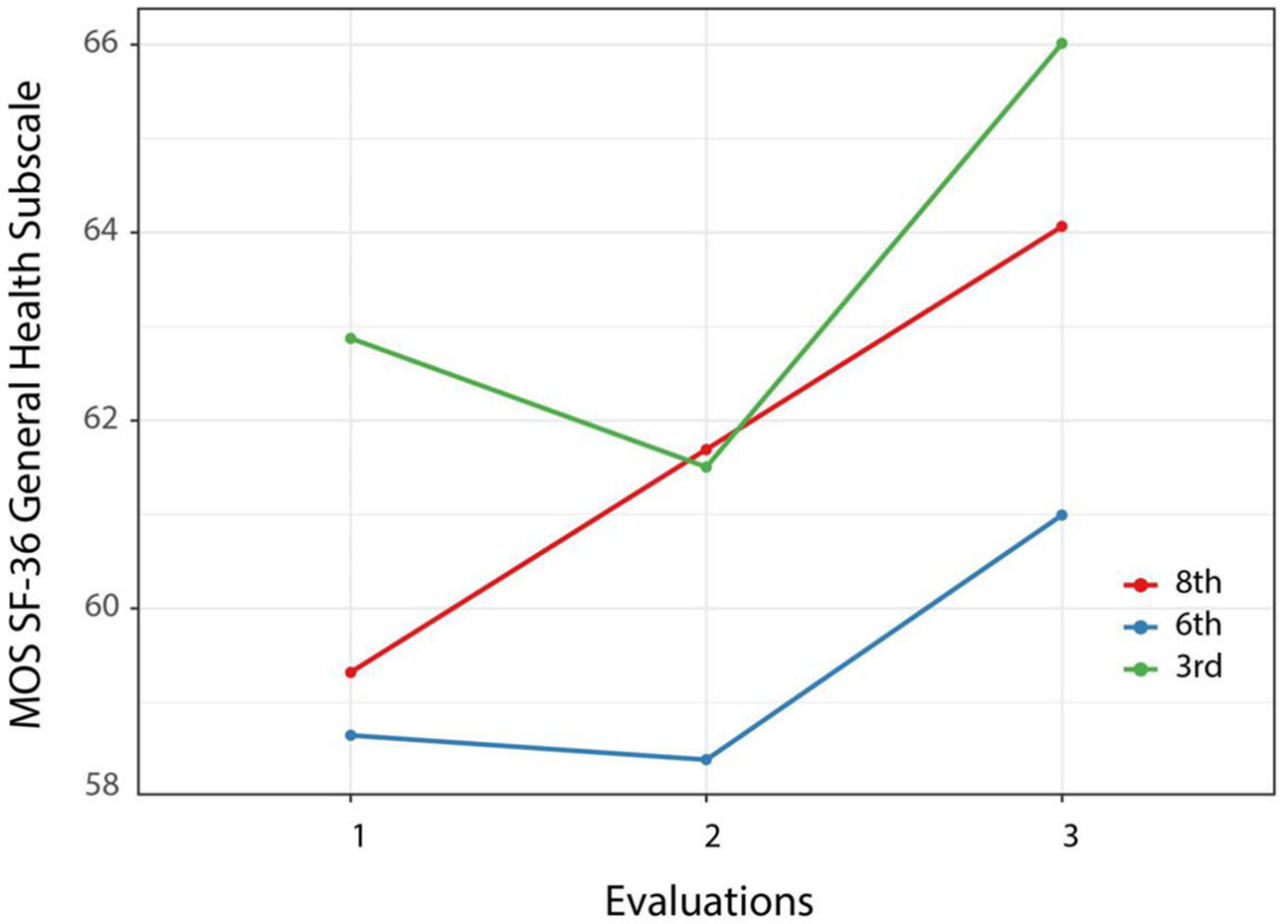
MOS SF-36 General Health subscale (HRQOL) means by group at each evaluation

The model for health-related quality of life (HRQOL assessed with the SF-36 General Health scale) is presented in Table 4. In this model, in addition to the effect of time, participants’ HRQOL was positively related to their level of education and inversely related to the number of health conditions they reported. Inspection of Figure 4 shows that the most pronounced effect on this outcome measure was observed at the three-month follow-up. Within group analyses showed that the 8th grade group improved significantly from baseline to the second follow-up (*t* [486] = 2.94, *p* = 0.01), while the 3rd grade group improved significantly only between the first and second follow-up (*t* [486) = 2.66, *p* = 0.02). Although the 6th grade group appears to have improved from the first to second follow-up, this change was not significant (*t* [475] = 1.61, *p* = 0.24. Calculation of treatment effect size through the likelihood ratio test of models resulted in significant chi square (χ2 [2] = 15.62, *p* < 0.001) for time, associated with a *d* value of 0.44 (95th CI 0.22-0.66), greater than the 0.20 but not reaching the more rigorous criterion of 0.50.

Finally, the model for self-reported medication adherence (Gonzalez-Lu factor score) is presented in Table 5. For this outcome, no overall effect for time was observed nor was there an interaction of time with treatment group. Both the 8th grade and 3rd grade groups reported significantly greater adherence at baseline (*t* [450] = 3.66, *p* < 0.001) and (t [456] = 3.67, *p* < 0.001), respectively. These group differences were maintained at the first follow-up, but only the 8th grade group was still significantly different from the 6th grade group at the second follow-up (*t* [516] = 2.52, *p* = 0.03). Although the 6th grade group appeared to improve adherence between the first and second follow-up, this improvement was not significant (*t* [477] = 2.07, *p* = 0.10). Calculation of treatment effect size through the likelihood ratio test resulted in nonsignificant chi square (χ2 [2] = 0.93, *p* = 0.63) for time, associated with a *d* value of 0.11 (95th CI −0.11-0.33), a small effect size that did not meet either criterion for an MCID [113].

## 4. Discussion

The purpose of this study was to investigate the impact of a tailored information app for CDSM in persons 40 years of age and older with chronic health conditions and low health literacy. The study focused on individuals 40 years of age and older because of the greater prevalence of chronic health conditions in persons in this age range. We hypothesized that the app would have a positive effect on four outcomes that reflect patients’ attitudes and beliefs about their ability to manage their health: activation, self-efficacy, HRQOL, and medication adherence. Results partially support this hypothesis, as we saw a significant positive impact of the app on three of the outcomes, activation, self-efficacy, and health-related quality of life that represented small to medium effect sizes consistent with MCID values from other studies. We did not find a significant change in participants’ self-report of medication adherence and it was not associated with an MCID. However, this interpretation must be advanced tentatively, as although changes in the outcome measures were observed over time, differences between experimental (3rd and 6th grade reading difficulty) and control conditions (8th grade reading level) were not observed making it difficult to determine whether the changes observed were the result of time along or participants’ interaction with the app.

Outcome measures for this study were chosen because of their relevance to middle aged and older person’s ability to manage their health and their use in previous studies, allowing us to compare results of the automated app with those of similar in-person and internet-delivered CDSM interventions. Patient activation was a key outcome and has been assessed in other studies in relation to disease self-management [111,112]. The effect sizes obtained in the present study are similar to those found in other studies of behavioral interventions to improve activation. In a meta-analysis of intervention studies aiming to improve activation of patients with chronic diseases, for example, Lin found average effects sizes of 0.33 for activation and 0.57 for self-efficacy, similar to our findings [111]. Other researchers, for example, have also found that CDSM programs can have a positive impact on self-efficacy for disease management [56,61,62] when compared to relevant control conditions.

A number of studies of behavioral interventions, however, have included HRQOL as an outcome, frequently finding positive effects of a CDSM intervention including a trial of delivering it via the internet [57]. Improved health-related quality of life was observed in this study as well, associated with a moderate effect size (0.50) that has been described as clinically meaningful for HRQOL outcomes in other areas [113].

In a similar vein, Ritter and Lorig [91], in a review of seven chronic disease self-management programs offered in small groups or by the Internet, report effect sizes ranging from 0.18 (a value that approaches the minimum effect size often considered to represent a MCID) to 0.61, a moderately large effect size that is larger than the stricter criterion for MCID of 0.50. They also report effect sizes from both in-person and Internet-delivered studies, with an average effect size of 0.41 for in-person and 0.28 for Internet studies [92] (Table 5, p. 1270). The effect sizes obtained in our study thus compares favorably with those in other studies which included control conditions consistent with an effect of the app on patient self-reports.

These results are thus similar to those observed in studies of in-person CDSM programs that have been shown to have a positive impact on activation [114], self-efficacy, and health-related quality of life. This suggests that some of the drawbacks to providing in-person CDSM interventions (cost, lack of trained personnel, accessibility) may be addressed by providing patients access to CDSM as a digital therapeutic. While it is true that the development of the mobile app was expensive initially, after initial deployment this sort of app can be inexpensive to maintain while providing access to large numbers of patients. We [115] and others [116] have shown that initial development and ongoing deployment costs can be substantially offset by benefits such as improved self-management behavior. Lindsay et al. [52], for example, showed that a substantial increase in activation was associated with lower follow-up costs, especially in high-risk populations.

Given the common critique of applications that do not provide patients information at the 3rd to 6th grade level, it is not clear why we did not find an effect of the modules’ text difficulty favoring the two experimental groups (3rd and 6th grade text) over the control (8th grade text). A possible explanation comes from research on educational applications in other situations in which researchers found a “contiguity effect” [117] in which learners presented text and graphics in close proximity, as was done in this study, resulted in superior learning compared to a condition in which graphic and text elements were separated. In more recent studies, research has shown that use of multimedia as an adjunct to traditional instruction may enhance comprehension and learner motivation [118]. Other research has shown that incorporating information technology in interventions may enhance patient engagement [119], an effect that was not controlled in our research design. Future research may help clarify whether multimedia instruction can help persons with low health literacy even when the text of the information presented is too difficult when presented as text alone.

In recruitment efforts for this study, we relied in part on several community organizations providing service for persons with low educational and economic resources. In the future, it might be possible to enlist their aid in supporting further deployment of the app, perhaps even including efforts to further develop interpersonal support and to crowdsource information about the problems of these individuals and the solutions they may have developed.

Limitations of this study include the nature of the sample, which was primarily Black, Indigenous, or Other People of Color (BIPOC), and differences between the two study sites on some variables. While we included variables on which the sites differed as covariates in statistical models as well as using site as an additional covariate, it is possible that the observed differences may have affected the study’s outcomes. On the other hand, in the search for persons with low health literacy, we succeeded in recruiting a large number of participants health literacy skills that might place them at a disadvantage in managing their health, and the study shows that the intervention was successful in increasing activation, self-efficacy, and HRQOL. The lack of between-site differences on outcome variables also supports the usefulness of the intervention, as it appears to have been efficacious in two diverse settings. A significant limitation is the lack of a finding on control vs experimental group outcomes. Although lack of differences related to reading difficulty is a concern, the clear effect of time suggests that the intervention had a significant impact on participants. Even without significant between-group differences, the alternative that participants improved in activation, self-efficacy, and HRQOL spontaneously over time is implausible. Additional research on possible mediators and moderators of change in these measures would be useful in further understanding these findings and enhancing the efficacy of the app. For example, the recruitment, screening, assessment, and intervention process provided substantial opportunities for personal interaction that may have affected outcomes. In the future it might be useful to clarify the extent to which intervention effects are related to personal interactions and intervention effects, although it should be noted that the effect sizes seen in the present study are similar to those of other studies that provided varying degrees of interpersonal contact. The fact that the intervention has only been tested with these groups is another limitation that may affect the generalizability of our findings. It should also be noted that although participants were assessed as having low health literacy on a standard measure, they had demonstrated some health-related skills as demonstrated by their having been diagnosed with a health condition. Finally, although in the development process we attempted to include potential users at all stages of development, even greater input from them might have resulted in different content for the app. Our focus on the broad effects of the intervention leaves open the question of whether it may have had differential effects related to specific diagnoses, an issue which may be further explored.

It should also be noted that the study included individuals 40 years of age and older because of their increased likelihood of experiencing chronic health conditions. There are important differences between individuals in their forties and those who are much older with respect to health literacy [2,120], history of educational opportunity [121], and use of digital health resources [122]. Further exploration of the effects of age within this group of persons aged 40 years and older may be useful in understanding how to make the app useful to individuals across this age range. This may be especially important given age-related issues in the acceptance and use of eHealth by middle aged and older adults [123].

## 5. Conclusions

In this study we investigated the effects of a mobile app for CDSM in persons aged 40 years and older with low levels of health literacy and chronic health conditions. Although we hypothesized that modules that presented content at the 8th grade level would be less effective than those at lower levels, this hypothesis was not supported. Change in self-report measures over time, however, suggest that the app may have had positive effects for all groups. Given the study design, it was not possible to assess the uptake of the app in a real-world situation. Future development of the app will include additional analyses of possible mediators and moderators of its effects to better understand how it works and ultimately have an even greater impact. Given the recent availability of generative artificial intelligence models, we plan to explore the potential to integrate interactive language capabilities in the app. Additional research will also focus on strategies for making the app widely available at little or no cost to users in order to increase uptake.

## Supplementary Material

Supplementary Material

## Figures and Tables

**Figure 5.**
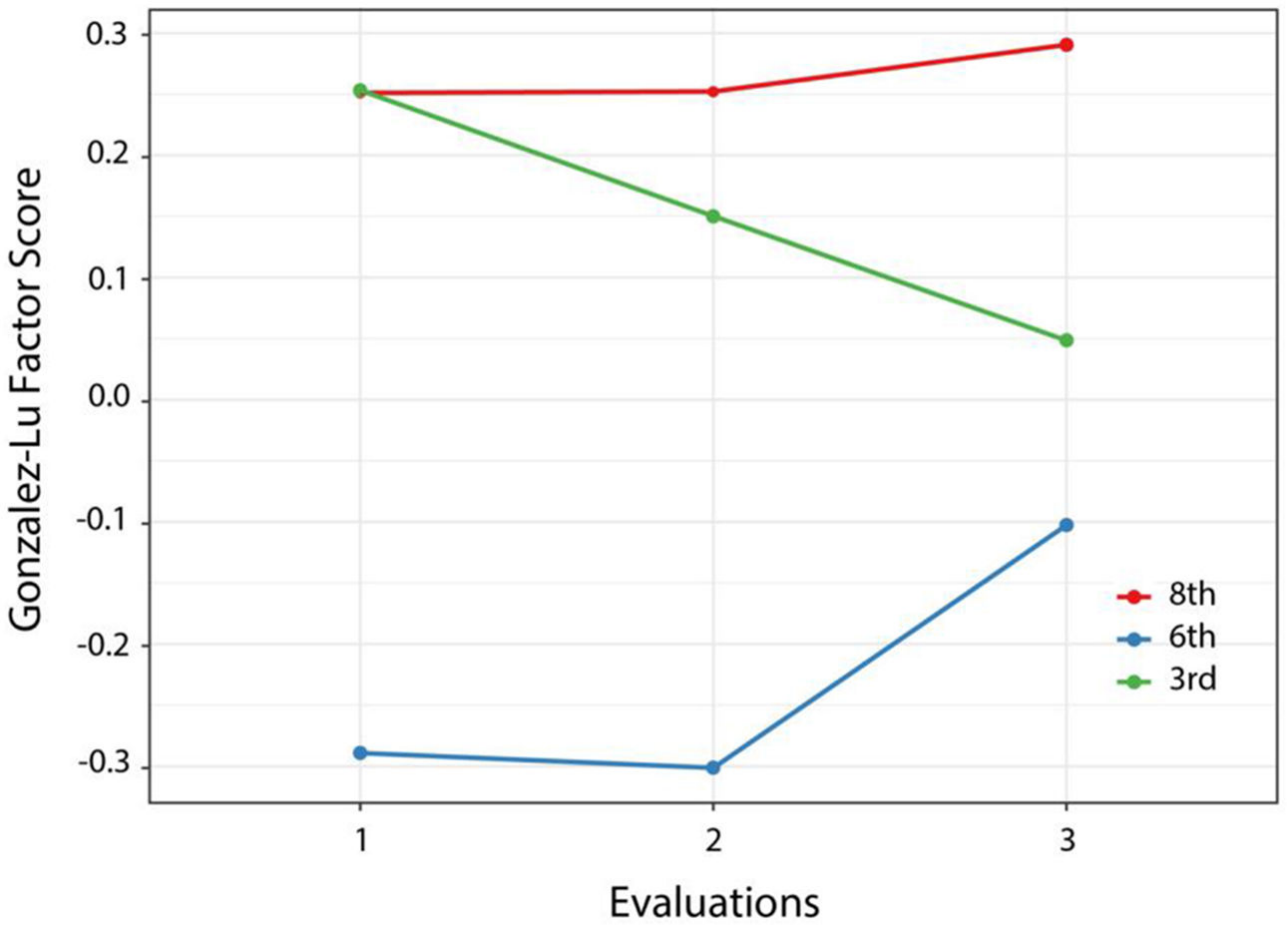
Gonzalez-Lu score means by group at each evaluation

**Table 1.** Description of participants completing at least one intervention session

Variable Count	Atlanta	Fort Lauderdale	Total	Χ^2^	df	*p*	Effect Size (*d*)
Men	65	79	144				
Women	118	47	165	22.15	1	< 0.001	0.56
White	7	34	41				
Nonwhite	176	92	268	37.78	1	< 0.001	0.71
Variable Mean (SD)				*t*	df	*p*	ES
Age in Years	58.10 (8.61)	56.95 (8.09)	57.63 (8.41)	1.18	307	0.24	0.14
Education Years	12.02 (1.76)	11.64 (1.97)	11.86 (1.85)	1.75	307	0.08	0.20
Total Number of Health Conditions	5.92 (2.66)	7.26 (2.77)	6.47 (2.78)	4.28	307	< 0.001	0.50
WJ Reading Grade^1^	6.60 (4.10)	7.74 (3.97)	7.06 (4.08)	2.44	307	0.02	0.28
Flight/Vidas Health Literacy^1^	9.59 (3.92)	10.97 (4.09)	10.17 (4.04)	2.89	307	0.004	0.35
PAM Score^1^	61.37 (16.03)	61.94 (15.96)	61.61 (15.97)	0.30	291^2^	0.76	0.04
CDSE Mean^1^	6.96 (2.01)	6.61 (1.93)	6.82 (1.98)	1.46	290^2^	0.15	0.18
HRQOL (SF General Health)^1^	60.21 (19.38)	60.16 (20.10)	60.19 (19.66)	0.02	288^2^	0.98	0.003
Gonzalez Lu Factor Score^1^	−0.09 (1.07)	0.14 (1.02)	0.01 (1.06)	1.89	291^2^	0.06	0.22

1 WJ Reading Grade = Woodcock-Johnson Psycho-Educational Battery Passage Comprehension subtest grade equivalent score; FV Health Literacy = FLIGHT-VIDAS Health Literacy Measure; PAM score = Patient Activation Measure; CDSE = Chronic Disease Self-Efficacy Scale; SF General Health = Medical Outcomes Studies, Short Form -36 General Health subscale; Gonzalez-Lu = Self-report of medication adherence factor score (see text); GL = Gonzalez-Lu questions 1-4.

2 Degrees of freedom vary due to data loss caused by equipment failure

**Table 2.** Model for the Patient Activation Measure

	Sum of Squares	Mean Squares	Numerator df	Denominator df	*F*	*p*
Age	601.86	601.86	1	281.16	4.90	***0.03***
Gender	394.9	394.9	1	274.52	3.21	0.07
Race	82.26	82.26	1	276.27	0.67	0.41
Education	143.46	143.46	1	270.92	1.17	0.28
Health Literacy	309.17	309.17	1	276.78	2.51	0.11
Health Conditions	189.27	189.27	1	281.6	1.54	0.22
Site	17.73	17.73	1	271.62	0.14	0.70
Time	822.22	411.11	2	482.77	3.34	***0.04***
Group	159	79.5	2	276.84	0.65	0.52
Time X Group	92.61	23.15	4	482.3	0.19	0.94

**Table 3.** Model for the Chronic Disease Self-Efficacy Scale

	Sum of Squares	Mean Squares	Numerator df	Denominator df	*F*	*p*
Age	1.20	1.17	1	291	0.90	0.34
Gender	1.30	1.31	1	285	1.01	0.32
Race	0.10	0.13	1	297	0.10	0.75
Education	14.60	14.58	1	279	11.24	***< 0.001***
Health Literacy	14.10	14.10	1	285	10.88	***0.0011***
Health Conditions	19.30	19.28	1	286	14.87	***< 0.001***
Site	0.60	0.62	1	287	0.48	0.49
Time	32.20	16.08	2	470	12.40	***< 0.001***
Group	3.80	1.92	2	287	1.48	0.23
Time X Group	7.60	1.91	4	470	1.47	0.21

**Table 4.** Model for MOS SF-36 General Health scale

	Sum of Squares	Mean Squares	Numerator df	Denominator df	*F*	*p*
Age	46	46	1	287	0.47	0.50
Gender	250	250	1	276	2.52	0.11
Race	13	13	1	276	0.13	0.72
Education	1139	1139	1	274	11.51	**< 0.001**
Health Literacy	259	259	1	277	2.62	0.11
Health Conditions	1739	1739	1	280	17.57	**< 0.001**
Site	172	172	1	274	1.74	0.19
Time	1558	779	2	474	7.87	**< 0.001**
Group	272	136	2	277	1.37	0.26
Time X Group	355	89	4	474	0.9	0.47

**Table 5.** Model for Gonzalez-Lu adherence score

	Sum of Squares	Mean Squares	Numerator df	Denominator df	*F*	*p*
Age	1.12	1.119	1	282	3.19	0.07
Gender	1.35	1.355	1	274	3.87	0.0503
Race	0.63	0.633	1	274	1.81	0.18
Education	0.01	0.008	1	270	0.02	0.88
Health Literacy	1.61	1.609	1	275	4.59	**0.03**
Health Conditions	0.94	0.94	1	280	2.68	0.10
Site	0.79	0.786	1	272	2.24	0.14
Time	0.28	0.138	2	476	0.39	0.67
Group	5.73	2.864	2	276	8.18	**<0.001**
Time X Group	3.09	0.772	4	476	2.2	0.07

## Data Availability

Data and statistical analysis syntax are available from the first author on request.

## References

[1] US Department of Health and Human Services. Healthy people 2030: Health literacy; Department of Health and Human Services. https://health.gov/healthypeople/priority-areas/social-determinants-health/literature-summaries/health-literacy Accessed 9/13/2023: Washington DC, 2023.

[2] Kutner M; Greenberg E; Jin Y; Paulsen C The health literacy of America’s adults: Results from the 2003 National Asessment of Adult Literacy (NCES 2006-483). US Department of Education, National Center for Educational Statistics: Washington, DC, 2006.

[3] OECD. OECD skills outlook 2013: First results from the survey of adults skills; OECD Publishing: Paris, 2013.

[4] National Center for Educational Statistics. US adults with low literacy and numeracy skills: 2012/2014 to 2017; National Center for Educational Statistics: Washington, DC, 2022.

[5] Baccolini V; Rosso A; Di Paolo C; Isonne C; Salerno C; Migliara G; Prencipe GP; Massimi A; Marzuillo C; De Vito C; What is the prevalence of low health literacy in European Union member states? A systematic review and meta-analysis. J Gen Intern Med 2021, 36, 753–761, doi:10.1007/s11606-020-06407-8. 33403622 PMC7947142

[6] Canadian Council on Learning. Health literacy in Canada: A healthy understanding; Canadian Council on Learning: Ottawa, 2008.

[7] Adekoya-Cole TO; Akinmokun OI; Enweluzo GO; Badmus OO; Alabi EO Poor health literacy in Nigeria: Causes, consequences and measures to improve it. Nig Q J Hosp Med 2015, 25, 112–117. 27295830

[8] Paasche-Orlow MK; Wolf MS Promoting health literacy research to reduce health disparities. J Health Commun 2010, 15 Suppl 2, 34–41, doi:926961883 [pii]; 10.1080/10810730.2010.499994 [doi]. 20845191

[9] Stormacq C; Van den Broucke S; Wosinski J Does health literacy mediate the relationship between socioeconomic status and health disparities? Integrative review. Health Promot Int 2019, 34, e1–e17, doi:10.1093/heapro/day062. 30107564

[10] Mantwill S; Monestel-Umana S; Schulz PJ The relationship between health literacy and health disparities: A systematic review. PLoS One 2015, 10, e0145455, doi:10.1371/journal.pone.0145455. 26698310 PMC4689381

[11] Pignone M; Dewalt DA; Sheridan S; Berkman N; Lohr KN Interventions to improve health outcomes for patients with low literacy. A systematic review. J Gen. Intern Med 2005, 20, 185–192, doi:JGI40208 [pii]; 10.1111/j.1525-1497.2005.40208.x [doi]. 15836553 PMC1490066

[12] Berkman ND; Sheridan SL; Donahue KE; Halpern DJ; Crotty K Low health literacy and health outcomes: an updated systematic review. Ann. Intern Med 2011, 155, 97–107, doi:155/2/97 [pii]; 10.1059/0003-4819-155-2-201107190-00005 [doi]. 21768583

[13] Bo A; Friis K; Osborne RH; Maindal HT National indicators of health literacy: ability to understand health information and to engage actively with healthcare providers - a population-based survey among Danish adults. BMC Public Health 2014, 14, 1095, doi:10.1186/1471-2458-14-1095. 25339154 PMC4286937

[14] Osborn CY; Cavanaugh K; Wallston KA; Kripalani S; Elasy TA; Rothman RL; White RO Health literacy explains racial disparities in diabetes medication adherence. J Health Commun 2011, 16 Suppl 3, 268–278, doi:10.1080/10810730.2011.604388 [doi]. 21951257 PMC3561717

[15] Osborn CY; Cavanaugh K; Wallston KA; White RO; Rothman RL Diabetes numeracy: an overlooked factor in understanding racial disparities in glycemic control. Diabetes Care 2009, 32, 1614–1619, doi:dc09-0425 [pii]; 10.2337/dc09-0425 [doi]. 19401443 PMC2732142

[16] Osborn CY; Paasche-Orlow MK; Bailey SC; Wolf MS The mechanisms linking health literacy to behavior and health status. Am. J Health Behav 2011, 35, 118–128, doi:10.5555/ajhb.2011.35.1.118 [pii]. 20950164 PMC3085858

[17] Osborn CY; Paasche-Orlow MK; Davis TC; Wolf MS Health literacy: An overlooked factor in understanding HIV health disparities. Am. J Prev. Med 2007, 33, 374–378, doi:S0749-3797(07)00465-5 [pii]; 10.1016/j.amepre.2007.07.022 [doi]. 17950402

[18] Waldrop-Valverde D; Osborn CY; Rodriguez A; Rothman RL; Kumar M; Jones DL Numeracy skills explain racial differences in HIV medication management. AIDS Behav 2010, 14, 799–806, doi:10.1007/s10461-009-9604-4 [doi]. 19669403 PMC2891293

[19] Rowlands G; Protheroe J; Winkley J; Richardson M; Seed PT; Rudd R A mismatch between population health literacy and the complexity of health information: An observational study. Br J Gen Pract 2015, 65, e379–386, doi:10.3399/bjgp15X685285. 26009533 PMC4439828

[20] Kessels RPC Patients' memory for medical information. Journal of the Royal Society of Medicine 2003, 96, 219–222. 12724430 10.1258/jrsm.96.5.219 PMC539473

[21] Kravitz RL; Hays RD; Sherbourne CD; DiMatteo MR; Rogers WH; Ordway L; Greenfield S Recall of recommendations and adherence to advice among patients with chronic medical conditions. Arch. Intern. Med 1993, 153, 1869–1878. 8250648

[22] Vinker S; Eliyahu V; Yaphe J The effect of drug information leaflets on patient behavior. Isr Med Assoc J 2007, 9, 383–386. 17591379

[23] Herber OR; Gies V; Schwappach D; Thurmann P; Wilm S Patient information leaflets: informing or frightening? A focus group study exploring patients' emotional reactions and subsequent behavior towards package leaflets of commonly prescribed medications in family practices. BMC Fam Pract 2014, 15, 163, doi:10.1186/1471-2296-15-163. 25277783 PMC4287479

[24] Kreuter M; Farrell D; Olevitch L; Brennan L Tailoring health messages; Lawrence Erlbaum: Mahwah, NJ, 2000.

[25] Schapira MM; Swartz S; Ganschow PS; Jacobs EA; Neuner JM; Walker CM; Fletcher KE Tailoring educational and behavioral interventions to level of health literacy: A systematic review. MDM Policy Pract 2017, 2, 2381468317714474, doi:10.1177/2381468317714474. 30288424 PMC6124923

[26] Taggart J; Williams A; Dennis S; Newall A; Shortus T; Zwar N; Denney-Wilson E; Harris MF A systematic review of interventions in primary care to improve health literacy for chronic disease behavioral risk factors. BMC Family Practice 2012, 13, 49, doi:10.1186/1471-2296-13-49. 22656188 PMC3444864

[27] Schillinger D; Duran ND; McNamara DS; Crossley SA; Balyan R; Karter AJ Precision communication: Physicians' linguistic adaptation to patients' health literacy. Science Advances 2021, 7, eabj2836, doi:doi:10.1126/sciadv.abj2836. 34919437 PMC8682984

[28] Ghalibaf AK; Nazari E; Gholian-Aval M; Tara M Comprehensive overview of computer-based health information tailoring: A systematic scoping review. BMJ Open 2019, 9, e021022, doi:10.1136/bmjopen-2017-021022. PMC6340008 30782671

[29] Lustria ML; Noar SM; Cortese J; Van Stee SK; Glueckauf RL; Lee J A meta-analysis of web-delivered tailored health behavior change interventions. J. Health Commun 2013, 18, 1039–1069, doi:10.1080/10810730.2013.768727 [doi]. 23750972

[30] Ownby RL; Acevedo A; Waldrop-Valverde D Enhancing the impact of mobile health literacy interventions to reduce health disparities. Quarterly Review of Distance Education 2019, 10, 15–34. PMC6752043 31537979

[31] Ginsburg GS; Phillips KA Precision medicine: From science to value. Health Aff (Millwood) 2018, 37, 694–701, doi:10.1377/hlthaff.2017.1624. 29733705 PMC5989714

[32] US Department of Health and Human Services. Healthy people 2020: Topics and objectives; Department of Health and Human Services. Available at http://healthypeople.gov/2020/topicsobjectives2020/pdfs/HP2020objectives.pdf. Accessed August 1, 2011: Washington DC, 2011.

[33] Centers for Disease Control. CDC clear communication index user guide; Centers for Disease Control: Atlanta GA, 2019.

[34] Protheroe J; Rowlands G Matching clinical information with levels of patient health literacy. Nurs Manag (Harrow) 2013, 20, 20–21, doi:10.7748/nm2013.06.20.3.20.e1095. 23841233

[35] Eltorai AE; Ghanian S; Adams CA Jr.; Born CT; Daniels AH Readability of patient education materials on the American Association for Surgery of Trauma website. Arch Trauma Res 2014, 3, e18161, doi:10.5812/atr.18161. 25147778 PMC4139691

[36] Hutchinson N; Baird GL; Garg M Examining the reading level of internet medical information for common internal medicine diagnoses. The American Journal of Medicine 2016, 129, 637–639, doi:10.1016/j.amjmed.2016.01.008. 26829438

[37] Miles RC; Baird GL; Choi P; Falomo E; Dibble EH; Garg M Readability of online patient educational materials related to breast lesions requiring surgery. Radiology 2019, 291, 112–118, doi:10.1148/radiol.2019182082. 30694156

[38] Rooney MK; Santiago G; Perni S; Horowitz DP; McCall AR; Einstein AJ; Jagsi R; Golden DW Readability of patient education materials from high-impact medical journals: A 20-year analysis. J Patient Exp 2021, 8, 2374373521998847, doi:10.1177/2374373521998847. 34179407 PMC8205335

[39] Ownby RL Readability of consumer-oriented geriatric depression information on the Internet. Clinical Gerontologist 2006, 29, 17–32, doi:10.1300/J018v29n04_02.

[40] Berkman ND; Sheridan SL; Donahue KE; Halpern DJ; Viera A; Crotty K; Holland A; Brasure M; Lohr KN; Harden E; Health literacy interventions and outcomes: an updated systematic review. Evidence report/technology assesment no. 199.; Agency for Healthcare Research and Quality: Rockville, MD, 2011. PMC4781058 23126607

[41] Lewis D Computer-based approaches to patient education: a review of the literature. J Am Med Inform. Assoc 1999, 6, 272–282. 10428001 10.1136/jamia.1999.0060272 PMC61369

[42] Lustria ML; Cortese J; Noar SM; Glueckauf RL Computer-tailored health interventions delivered over the Web: Review and analysis of key components. Patient. Educ. Couns 2009, 74, 156–173, doi:S0738-3991(08)00469-2 [pii]; 10.1016/j.pec.2008.08.023 [doi]. 18947966

[43] Kuhn E; Weiss BJ; Taylor KL; Hoffman JE; Ramsey KM; Manber R; Gehrman P; Crowley JJ; Ruzek JI; Trockel M CBT-I Coach: A description and clinician perceptions of a mobile app for cognitive behavioral therapy for insomnia. Journal of clinical sleep medicine 2016, 12, 597–606, doi:10.5664/jcsm.5700. 26888586 PMC4795288

[44] McNaughton JL Brief interventions for depression in primary care: a systematic review. Can. Fam Physician 2009, 55, 789–796, doi:55/8/789 [pii]. 19675262 PMC2726093

[45] Hibbard JH; Stockard J; Mahoney ER; Tusler M Development of the Patient Activation Measure (PAM): conceptualizing and measuring activation in patients and consumers. Health Serv. Res 2004, 39, 1005–1026, doi:10.1111/j.1475-6773.2004.00269.x [doi];HESR269 [pii]. 15230939 PMC1361049

[46] Greene J; Hibbard JH Why does patient activation matter? An examination of the relationships between patient activation and health-related outcomes. J Gen Intern Med 2012, 27, 520–526, doi:10.1007/s11606-011-1931-2. 22127797 PMC3326094

[47] Hosseinzadeh H; Verma I; Gopaldasani V Patient activation and Type 2 diabetes mellitus self-management: A systematic review and meta-analysis. Australian Journal of Primary Health 2020, 26, 431–442, doi:10.1071/PY19204. 33222755

[48] Mosen DM; Schmittdiel J; Hibbard J; Sobel D; Remmers C; Bellows J Is patient activation associated with outcomes of care for adults with chronic conditions? The Journal of Ambulatory Care Management 2007, 30, 21–29. 17170635 10.1097/00004479-200701000-00005

[49] Hibbard JH; Greene J What the evidence shows about patient activation: Better health outcomes and care experiences; fewer eata on costs. Health Affairs 2013, 32, 207–214, doi:10.1377/hlthaff.2012.1061. 23381511

[50] Greene J; Hibbard J; Tusler M How much do health literacy and patient activation contribute to older adults’ ability to manage their health?; AARP. Available at: http://assets.aarp.org/rgcenter/health/2005_05_literacy.pdf: Washington, DC, 2005.

[51] Greene J; Hibbard JH; Sacks R; Overton V; Parrotta CD When patient activation levels change, health outcomes and costs change, too. Health Aff (Millwood) 2015, 34, 431–437, doi:10.1377/hlthaff.2014.0452. 25732493

[52] Lindsay A; Hibbard JH; Boothroyd DB; Glaseroff A; Asch SM Patient activation changes as a potential signal for changes in health care costs: Cohort study of US high-cost patients. Journal of General Internal Medicine 2018, 33, 2106–2112, doi:10.1007/s11606-018-4657-6. 30291604 PMC6258627

[53] Bandura A The primacy of self-regulation in health promotion. Applied Psychology 2005, 54, 245–254, doi:10.1111/j.1464-0597.2005.00208.x.

[54] Bandura A Health promotion from the perspective of social cognitive theory. In Proceedings of the Psychology & Health, 7/1/1998, 1998; pp. 623–649.

[55] Bodenheimer T; Lorig K; Holman H; Grumbach K Patient self-management of chronic disease in primary care. JAMA 2002, 288, 2469–2475. 12435261 10.1001/jama.288.19.2469

[56] Lorig KR; Ritter P; Stewart AL; Sobel DS; Brown BW Jr.; Bandura A; Gonzalez VM; Laurent DD; Holman HR Chronic disease self-management program: 2-year health status and health care utilization outcomes. Med Care 2001, 39, 1217–1223. 11606875 10.1097/00005650-200111000-00008

[57] Lorig KR; Ritter PL; Laurent DD; Plant K Internet-based chronic disease self-management: A randomized trial. Med Care 2006, 44, 964–971, doi:10.1097/01.mlr.0000233678.80203.c1 [doi];00005650-200611000-00002 [pii]. 17063127

[58] Chen J; Tian Y; Yin M; Lin W; Tuersun Y; Li L; Yang J; Wu F; Kan Y; Li X; Relationship between self-efficacy and adherence to self-management and medication among patients with chronic diseases in China: A multicentre cross-sectional study. Journal of Psychosomatic Research 2023, 164, 111105, doi:10.1016/j.jpsychores.2022.111105. 36495756

[59] Hoong JM; Koh HA; Wong K; Lee HH Effects of a community-based chronic disease self-management programme on chronic disease patients in Singapore. Chronic Illness 0, 17423953221089307, doi:10.1177/17423953221089307. 35317664

[60] Farley H Promoting self-efficacy in patients with chronic disease beyond traditional education: A literature review. Nurs Open 2020, 7, 30–41, doi:10.1002/nop2.382. 31871689 PMC6917929

[61] Lorig KR; Sobel DS; Ritter PL; Laurent D; Hobbs M Effect of a self-management program on patients with chronic disease. Eff Clin Pract 2001, 4, 256–262. 11769298

[62] Ory MG; Ahn S; Jiang L; Smith ML; Ritter PL; Whitelaw N; Lorig K Successes of a national study of the Chronic Disease Self-Management Program: Meeting the triple aim of health care reform. Med Care 2013, 51, 992–998, doi:10.1097/MLR.0b013e3182a95dd1. 24113813

[63] Fernandez-Lazaro CI; García-González JM; Adams DP; Fernandez-Lazaro D; Mielgo-Ayuso J; Caballero-Garcia A; Moreno Racionero F; Córdova A; Miron-Canelo JA Adherence to treatment and related factors among patients with chronic conditions in primary care: A cross-sectional study. BMC Family Practice 2019, 20, 132, doi:10.1186/s12875-019-1019-3. 31521114 PMC6744672

[64] DiMatteo MR Variations in patients' adherence to medical recommendations: A quantitative review of 50 years of research. Med Care 2004, 42, 200–209, doi:10.1097/01.mlr.0000114908.90348.f9. 15076819

[65] Walsh CA; Cahir C; Tecklenborg S; Byrne C; Culbertson MA; Bennett KE The association between medication non-adherence and adverse health outcomes in ageing populations: A systematic review and meta-analysis. British Journal of Clinical Pharmacology 2019, 85, 2464–2478, doi:10.1111/bcp.14075. 31486099 PMC6848955

[66] Cutler RL; Fernandez-Llimos F; Frommer M; Benrimoj C; Garcia-Cardenas V Economic impact of medication non-adherence by disease groups: A systematic review. BMJ Open 2018, 8, e016982, doi:10.1136/bmjopen-2017-016982. PMC5780689 29358417

[67] Ownby RL; Acevedo A; Waldrop-Valverde D; Caballero J; Simonson M; Davenport R; Kondwani K; Jacobs RJ A mobile app for chronic disease self-management: Protocol for a randomized controlled trial. JMIR Res Protoc 2017, 6, e53, doi:10.2196/resprot.7272. 28381395 PMC5399224

[68] Lorig K; Ritter PL; Ory MG; Whitelaw N Effectiveness of a generic chronic disease self-management program for people with type 2 diabetes: A translation study. Diabetes Educ 2013, doi:0145721713492567 [pii]; 10.1177/0145721713492567 [doi]. 23782621

[69] Jaglal S; Guilcher S; Hawker G; Lou W; Salbach N; Manno M; Zwarenstein M Impact of a chronic disease self-management program on health care utilization in rural communities: a retrospective cohort study using linked administrative data. BMC Health Services Research 2014, 14, 198, doi:10.1186/1472-6963-14-198. 24885135 PMC4036726

[70] Jacobs RJ; Ownby RL; Acevedo A; Waldrop-Valverde D A qualitative study examining health literacy and chronic illness self-management in Hispanic and non-Hispanic older adults. J Multidiscip Healthc 2017, 10, 167–177, doi:10.2147/JMDH.S135370. 28461754 PMC5404800

[71] Mayer RE Multimedia learning (2nd ed.); Cambridge: New York, 2009.

[72] Patel N; Waldrop D; Ownby RL Creating a tailored info app to promote self-management skills in persons with chronic health conditions: Development strategies and user experience [preprint] Distance Learning (Greenwich) 2023, 20, 9–18. PMC10911517 38440090

[73] Fry E A readability formula that saves time. Journal of Reading 1968, 11, 513–578.

[74] Flesch R A new readability yardstick. Journal of Applied Psychology 1948, 32, 221–233. 18867058 10.1037/h0057532

[75] Kincaid JP; Fishburne RP; Rogers RL; Chissom BS Derivation of new readability formulas (Automated Readability Index, Fog Count, and Flesch Reading Ease Formula) for Navy enlisted personnel; Chief of Naval Technical Training. Available online at http://www.dtic.mil/dtic/tr/fulltext/u2/a006655.pdf.: Millington, TN, 1975.

[76] National Institutes of Health Staff. What are the requirements for reporting on human study participant age, sex/gender, and race/ethnicity? Available online: https://nexus.od.nih.gov/all/2021/04/29/what-are-the-requirements-for-reporting-on-human-study-participant-age-sex-gender-and-race-ethnicity/ (accessed on March 28, 2023).

[77] Murphy PW; Davis TC; Long SW; Jackson RH; Decker BC Rapid Estimate of Adult Literacy in Medicine (REALM): A quick reading test for patients. Journal of Reading 1993, 37, 124–130.

[78] Han HJ; Acevedo A; Waldrop-Valverde D; Ownby RL Evaluation of short forms of the Rapid Estimate of Adult Literacy in Medicine (REALM). In Proceedings of the International Conference on Communication in Healthcare/Health Literacy Annual Researach Conference, Baltimore MD, October, 2017, 2017, October.

[79] Fried VM; Bernstein AB; Bush MA Multiple chronic conditions among adults aged 45 and over: Trends over the past 10 years. NCHS data brief, no. 100; National Center for Health Statistics: Hyattsville, MD, 2012. 23101759

[80] Solé-Auró A; Michaud P-C; Hurd M; Crimmins E Disease incidence and mortality among older Americans and Europeans. Demography 2015, 52, 593–611, doi:10.1007/s13524-015-0372-7. 25715676 PMC4441205

[81] Tinetti ME; Fried TR; Boyd CM Designing health care for the most common chronic condition--multimorbidity. JAMA 2012, 307, 2493–2494, doi:1187936 [pii]; 10.1001/jama.2012.5265 [doi]. 22797447 PMC4083627

[82] Groll DL; To T; Bombardier C; Wright JG The development of a comorbidity index with physical function as the outcome. Journal of clinical epidemiology 2005, 58, 595–602. 15878473 10.1016/j.jclinepi.2004.10.018

[83] Centers for Medicare and Medicaid Services. Chronic conditions among Medicare beneficiaries, chartbook (2012 ed.). Centers for Medicare and Medicaid Services: Baltimore, MD, 2012.

[84] Diederichs C; Berger K; Bartels DB The measurement of multiple chronic diseases—a systematic review on existing multimorbidity indices. The Journals of Gerontology: Series A 2010, 66A, 301–311, doi:10.1093/gerona/glq208. 21112963

[85] Woodcock RW; McGrew KS; Mather N Woodcock-Johnson III Normative Update; Riverside: Rolling Meadows, IL, 2007.

[86] Ownby RL; Acevedo A; Waldrop-Valverde D; Jacobs RJ; Homs AM; Czaja SJ; Loewenstein D Development and initial validation of a computer-administered health literacy assessment in Spanish and English: FLIGHT/VIDAS. Patient Related Outcome Measures 2013, 4, 1–15. 23990736 10.2147/PROM.S48384 PMC3753170

[87] Pleasant A Advancing health literacy measurement: a pathway to better health and health system performance. J. Health Commun 2014, 19, 1481–1496, doi:10.1080/10810730.2014.954083 [doi]. 25491583 PMC4292229

[88] Hibbard JH; Mahoney ER; Stockard J; Tusler M Development and testing of a short form of the Patient Activation Measure. Health Serv Res 2005, 40, 1918–1930, doi:10.1111/j.1475-6773.2005.00438.x. 16336556 PMC1361231

[89] Skolasky RL; Green AF; Scharfstein D; Boult C; Reider L; Wegener ST Psychometric properties of the patient activation measure among multimorbid older adults. Health Serv Res 2011, 46, 457–478, doi:10.1111/j.1475-6773.2010.01210.x. 21091470 PMC3064914

[90] Lorig K; Stewart A; Ritter P; Gonzalez VM; Laurent D; Lynch J Outcome measures for health education and other health care interventions; Sage: Thousand Oaks CA, 1996.

[91] Ritter PL; Lorig K The English and Spanish Self-Efficacy to Manage Chronic Disease Scale measures were validated using multiple studies. Journal of Clinical Epidemiology 2014, 67, 1265–1273, doi:10.1016/j.jclinepi.2014.06.009. 25091546

[92] Ware JE; Sherbourne CD The MOS 36-item short-form health survey (SF-36). I. Conceptual framework and item selection. Med Care 1992, 30, 478–483. 1593914

[93] Peeters G; Waller M; Dobson AJ SF-36 normative values according to level of functioning in older women. Quality of Life Research 2019, 28, 979–989, doi:10.1007/s11136-018-2077-z. 30511256

[94] Lyons RA; Perry IM; Littlepage BNC Evidence for the validity of the Short-form 36 Questionnaire (SF-36) in an elderly population. Age and Ageing 1994, 23, 182–184, doi:10.1093/ageing/23.3.182. 8085500

[95] McHorney CA; Ware JE Jr.; Lu JF; Sherbourne CD The MOS 36-item Short-Form Health Survey (SF-36): III. Tests of data quality, scaling assumptions, and reliability across diverse patient groups. Med Care 1994, 32, 40–66. 8277801 10.1097/00005650-199401000-00004

[96] Gonzalez JS; Schneider HE; Wexler DJ; Psaros C; Delahanty LM; Cagliero E; Safren SA Validity of medication adherence self-reports in adults with type 2 diabetes. Diabetes Care 2013, 36, 831–837, doi:dc12-0410 [pii]; 10.2337/dc12-0410 [doi]. 23204245 PMC3609536

[97] Lu M; Safren SA; Skolnik PR; Rogers WH; Coady W; Hardy H; Wilson IB Optimal recall period and response task for self-reported HIV medication adherence. AIDS Behav 2008, 12, 86–94, doi:10.1007/s10461-007-9261-4 [doi]. 17577653

[98] McNeish D; Wolf MG Thinking twice about sum scores. Behavior Research Methods 2020, 52, 2287–2305, doi:10.3758/s13428-020-01398-0. 32323277

[99] Ferrando PJ; Lorenzo-Seva U On the added value of multiple factor score estimates in essentially unidimensional models. Educ Psychol Meas 2019, 79, 249–271, doi:10.1177/0013164418773851. 30911192 PMC6425092

[100] Horton JC; Jones MR Warning on inaccurate Rosenbaum cards for testing near vision. Surv Ophthalmol 1997, 42, 169–174, doi:10.1016/s0039-6257(97)00055-6. 9381371

[101] R Core Team. R: A language and environment for statistical computing.; R Foundation for Statistical Computing: Vienna, Austria, 2022.

[102] Bates D; Maechler M; Bolker B; Walker S Fitting linear mixed-effects models using lme4. Journal of Statistical Software 2015, 67, 1:48, doi:10.18637/jss.v067.i01.

[103] Luke SG Evaluating significance in linear mixed-effects models in R. Behav Res Methods 2017, 49, 1494–1502, doi:10.3758/s13428-016-0809-y. 27620283

[104] Lenth R; . emmeans: Estimated Marginal Means, aka Least-Squares Means. R package version 1.8.7. 2023, https://cran.r-project.org/web/packages/emmeans/index.html.

[105] Hintze J PASS 16; NCSS, LLC.: Kaysville UT, 2018.

[106] Ownby RL; Waldrop-Valverde D; Caballero J; Jacobs RJ Baseline medication adherence and response to an electronically delivered health literacy intervention targeting adherence. Neurobehav HIV Med 2012, 4, 113–121, doi:10.2147/NBHIV.S36549 [doi]. 23293544 PMC3535445

[107] Cohen J Statistical power analysis for the behavioral sciences (2nd ed.); Routledge: New York, 1988.

[108] Ludecke D esc: Effect size computation for meta analysis (version 0.5.1). 2019, doi:10.5281/zenodo.1249218.

[109] Mouelhi Y; Jouve E; Castelli C; Gentile S How is the minimal clinically important difference established in health-related quality of life instruments? Review of anchors and methods. Health and Quality of Life Outcomes 2020, 18, 136, doi:10.1186/s12955-020-01344-w. 32398083 PMC7218583

[110] Norman GR; Sloan JA; Wyrwich KW Interpretation of changes in health-related quality of life: The remarkable universality of half a standard deviation. Med Care 2003, 41, 582–592, doi:10.1097/01.MLR.0000062554.74615.4C. 12719681

[111] Lin M-Y; Weng W-S; Apriliyasari RW; Van Truong P; Tsai P-S Effects of patient activation intervention on chronic diseases: A meta-analysis. Journal of Nursing Research 2020, 28. 10.1097/jnr.0000000000000387 32649394

[112] Innab A; Kerari A Impact of behavioral interventions on patient activation in adults with hypertension: A systematic review and meta-analysis. Inquiry 2022, 59, 469580221090408, doi:10.1177/00469580221090408. 35635036 PMC9152571

[113] Sloan JA; Dueck A Issues for statisticians in conducting analyses and translating results for quality of life end points in clinical trials. J Biopharm Stat 2004, 14, 73–96, doi:10.1081/BIP-120028507. 15027501

[114] Gholami M; Abdoli Talaei A; Tarrahi MJ; Mirzaei Taqi F; Galehdar N; Pirinezhad P The effect of self-management support program on patient activation and inner strength in patients with cardiovascular disease. Patient Educ Couns 2021, 104, 2979–2988, doi:10.1016/j.pec.2021.04.018. 33972129

[115] Ownby RL; Waldrop-Valverde D; Jacobs RJ; Acevedo A; Caballero J Cost effectiveness of a computer-delivered intervention to improve HIV medication adherence. BMC Medical Informatics and Decision Making 2013, 13, 29. 23446180 10.1186/1472-6947-13-29 PMC3599639

[116] Gentili A; Failla G; Melnyk A; Puleo V; Tanna GLD; Ricciardi W; Cascini F The cost-effectiveness of digital health interventions: A systematic review of the literature. Frontiers in Public Health 2022, 10, doi:10.3389/fpubh.2022.787135. PMC9403754 36033812

[117] Moreno R; Mayer RE Cognitive principles of multimedia learning: The role of modality and contiguity. Journal of Educational Psychology 1999, 91, 358–368, doi:10.1037/0022-0663.91.2.358.

[118] Klimova B; Zamborova K Use of mobile applications in developing reading comprehension in second language acquisition--a review study. Education Sciences 2020, 10, 391, doi:10.3390/educsci10120391.

[119] Sawesi S; Rashrash M; Phalakornkule K; Carpenter JS; Jones JF The impact of information technology on patient engagement and health behavior change: A systematic review of the literature. JMIR Med Inform 2016, 4, e1, doi:10.2196/medinform.4514. 26795082 PMC4742621

[120] Rikard RV; Thompson MS; McKinney J; Beauchamp A Examining health literacy disparities in the United States: A third look at the National Assessment of Adult Literacy (NAAL). BMC Public Health 2016, 16, 975, doi:10.1186/s12889-016-3621-9. 27624540 PMC5022195

[121] Ryan CL; Bauman K Educational attainment in the United States: 2015; US Census Bureau: Suitland, MD, 2016.

[122] Chen C; Ding S; Wang J Digital health for aging populations. Nature Medicine 2023, 29, 1623–1630, doi:10.1038/s41591-023-02391-8. 37464029

[123] Wilson J; Heinsch M; Betts D; Booth D; Kay-Lambkin F Barriers and facilitators to the use of e-health by older adults: a scoping review. BMC Public Health 2021, 21, 1556, doi:10.1186/s12889-021-11623-w. 34399716 PMC8369710

