# Supplementary material for: A mobile app for chronic disease self-management for individuals with low health literacy: A multisite randomized controlled clinical trial
Source: J Ageing Longev. Author manuscript; Available in PMC 2024 Nov 15. (PMC11567679; doi:10.3390/jal4020005)
Supplement: Supplementary Material [file NIHMS1992002-supplement-Supplementary_Material.docx]

**Ownby et al.,** **“A mobile app for chronic disease self-management for individuals with low health literacy: A multisite randomized controlled clinical trial,” supplementary materials**

**Supplementary Table 1**

**Content overview of chronic disease self-management modules**

| **Title** | **Topics** |
| --- | --- |
| **Session 1** |  |
| **Introduction** | Goals of the course; definition of chronic health conditions and the kinds of problems they create. How the participants can become more involved in self-management and become a more active partner with their healthcare providers |
|  | Self-management of problems that are common in multimorbidity, such as fatigue, sleep problems, shortness of breath, and pain. |
|  | Definition of self-efficacy and how it can affect health; relation of self-efficacy to stress and coping; how to increase self-efficacy |
|  | Overview of module topics to be covered: Fatigue, Pain, Shortness of Breath, Sleep, Mood, Anger, Stress, Working with Providers, |
|  | Definition of self-management and how information in the modules can help develop self-management skills. |
|  | Cognitive behavioral model: Using your mind to feel better |
|  | Creating action plans |
| **Adherence (content tailored to level of depressive symptoms as assessed by the CES-D^1^)** | Goal: Helping participant to take charge of their health |
|  | Questions on emotional support, medication adherence, and provider relation |
|  | Healthy living |
|  | Self-monitoring |
|  | Strategies for medication adherence: schedule, pillboxes |
|  | Making a list of questions for next visit with provider, “Ask Me 3”^2^ |
|  | Impact of depression on adherence and feedback on mood from CES-D |
|  | Becoming active in own healthcare; use of internet and how to judge websites; healthy living |
| **Stress** | Goal, definition |
|  | Biology of stress |
|  | Stress effects on the body, including worsening some health conditions |
|  | Stress management: relaxation, meditation, goal setting and time management |
| **Session 2** |  |
| **Sleep (content tailored to level of depressive symptoms as assessed by the CES-D^1^ and stress by the PSS^3^)** | Purpose; association of chronic conditions and sleep problems |
|  | Basic information about sleep, including sleep stages and relation of sleep to mood and energy |
|  | Rules for better sleep: Sleep hygiene recommendations |
|  | Sleep diary |
|  | Creating action plan |
| **Mood (content tailored to level of depressive symptoms as assessed by CES-D^1^)** | Goal |
|  | Effects of depression on energy, mental functioning, attitudes toward self-care |
|  | Things that can worsen depression: stress, life events, alcohol and other substance use; some medical conditions |
|  | Cognitive behavioral approach to depression, identifying automatic thoughts, activity scheduling |
|  | Treatments for depression: medications, psychotherapy |
|  | Creating action plan |
| **Pain** | Goal |
|  | What is pain? Acute vs chronic pain |
|  | Factors that may exacerbate |
|  | Medications and substance use |
|  | Strategies for pain management, including working with provider on medication, physical activity, understanding the impact of mood on pain |
|  | Creating an action plan |
| **Memory** | Purpose |
|  | Memory Self-Efficacy Scale |
|  | How memory works; acquisition, storage, retrieval |
|  | Cognitive aging and things that can help memory, such as good sleep, exercise, mood; external memory aids |
| **Session 3** |  |
| **Fatigue** | Definition of fatigue, not merely tiredness; effects of fatigue on daily functioning |
|  | Managing fatigue: regular schedules, alternative treatments, self-help groups, counseling, learning more about fatigue, limited exercise (caution about CFS/ME patients) |
|  | Self-monitoring, tracking good days and bad, evaluating the relation between activity and fatigue |
| **Shortness of Breath** | Goal and definition |
|  | Anatomy of breathing, muscles involved in breathing |
|  | Consult with doctor; consider gradual increase in physical activity to reduce deconditioning |
|  | Do not smoke |
|  | Use medications, including inhalers, as directed |
|  | Breathing strategies: pursed lips and diaphragmatic breathing |
| **Anger** | Purpose |
|  | Mental processes related to anger |
|  | Things that make it worse: poor sleep, hunger, pain |
|  | Recognizing triggers and escalation |
|  | Anger, frustration, and chronic health conditions |
|  | Self-management strategies, avoiding triggers and escalation |
|  | Relaxation, deep breathing, self-talk, reframing |
|  | Avoiding alcohol and other substances |
|  | Getting professional help |

**Supplementary Figure 1:**

**Figure 1. Intervention screen encouraging healthy lifestyle**

Note: Adobe® product screenshot reprinted with permission from Adobe.


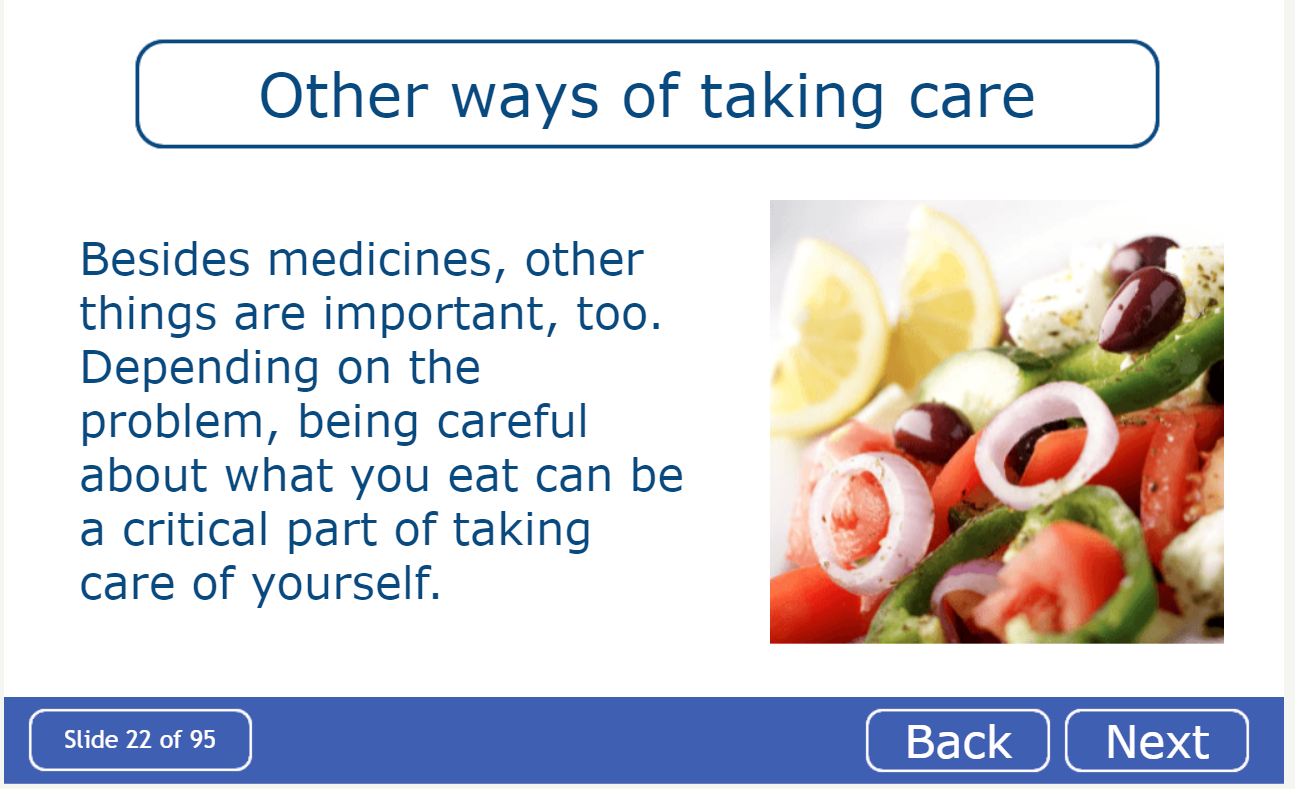


Supplementary Figure 2:


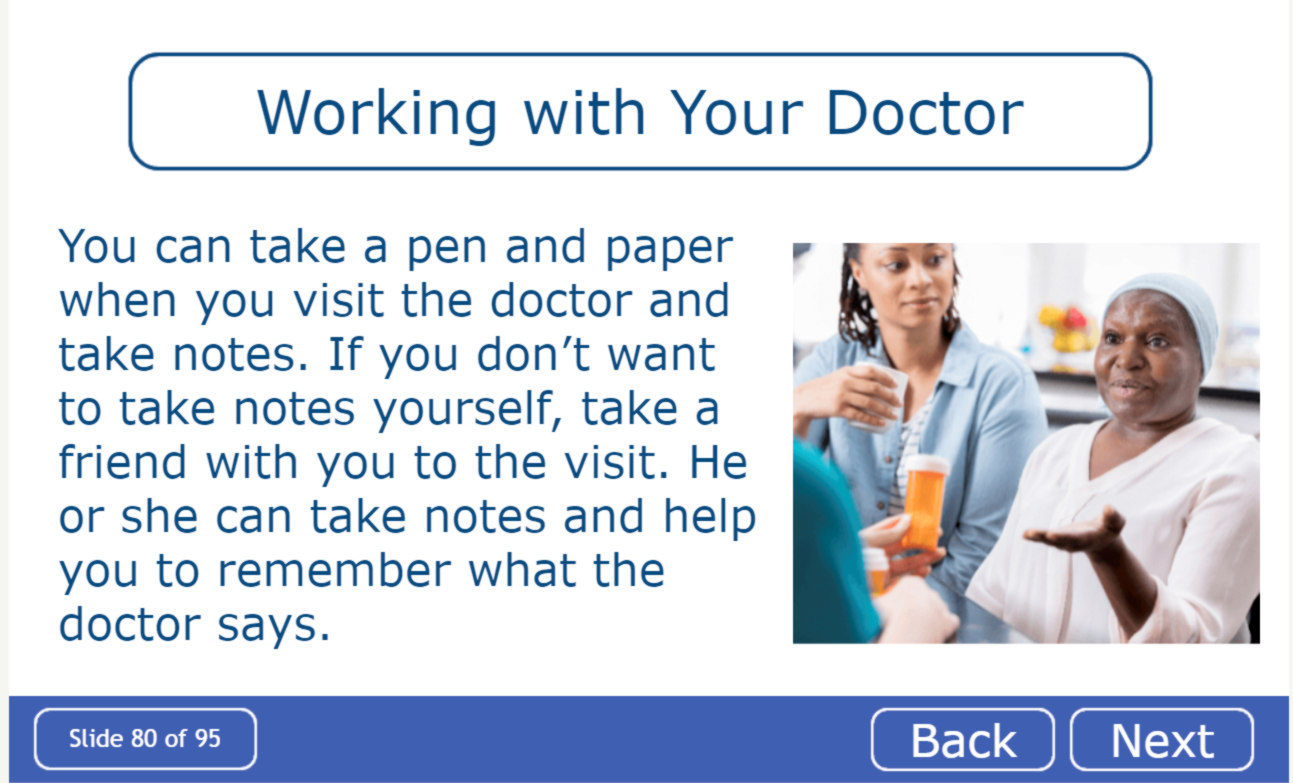


**Figure 2. Intervention screen showing suggestions for working with healthcare provider**

Note: Adobe® product screenshot reprinted with permission from Adobe.

References

1. Radloff LS. The CES-D Scale: A self-report depression scale for research in the general population. *Applied Psychological Measurement* 1977; 1: 385-401.

2. Institute for Healthcare Improvement. Ask Me 3 Brochure, <https://www.ihi.org/resources/Pages/Tools/Ask-Me-3-Good-Questions-for-Your-Good-Health.aspx> (undated).

3. Cohen S, Kamarck T and Mermelstein R. A global measure of perceived stress. *Journal of Health and Social Behavior* 1983; 24: 385-396.
